# Development, Characterization, and Stability of Burgers With Partial or Total Replacement of Beef by Vegetable Ingredients

**DOI:** 10.1111/1750-3841.71217

**Published:** 2026-06-20

**Authors:** Sheyla Maria Barreto Amaral, Silmara de Fátima Silva Cavalcante, Janevane Silva de Castro, Luiz Alves Bitu, Felipe Sousa da Silva, Mairlane Silva de Alencar, Adriano Lincoln Albuquerque Mattos, Ana Erbênia Pereira Mendes, Paulo Henrique Machado de Sousa, Elisabeth Mary Cunha da Silva

**Affiliations:** ^1^ Postgraduate Program in Food Science and Technology Federal University of Ceará Fortaleza Ceará Brazil; ^2^ Department of Food Engineering Center of Agrarian Sciences Federal University of Ceará Fortaleza Ceará Brazil; ^3^ Instrumental Chemistry Laboratory Center for Technology and Industrial Quality of Ceará Fortaleza Ceará Brazil; ^4^ Agroindustrial Process Laboratories Brazilian Agricultural Research Corporation Fortaleza Ceará Brazil; ^5^ Cultura and Art Institute Federal University of Ceará Fortaleza Ceará Brazil

**Keywords:** Analog plant‐based, Antioxidant, *Euterpe oleracea* Mart, Flexitarianism, hybrid products

## Abstract

This study evaluated the partial or total replacement of beef (0%, 25%, 50%, 75%, and 100%) with plant‐based ingredients (risotto rice, textured soy protein, and cassava starch) in burgers, combined with the incorporation of freeze‐dried açaí pulp as an antioxidant and natural colorant, and sesame oil as a substitute for beef fat. The effects of these substitutions on physicochemical, chemical, and physical properties, as well as the stability during 120 days of frozen storage, were investigated. Increased levels of meat replacement reduced protein content but improved technological performance, with less cooking loss and shrinkage and higher yield. The 50% replacement formulation showed no significant differences (*p* ≥ 0.05) from the 100% beef control in morphology, hardness, gumminess, and chewiness attributes. Higher replacement levels (75% and 100%) further increased yield but also led to greater structural changes. Freeze‐dried açaí pulp contributed to a reddish‐purple color. It maintained pH during storage, although conclusions regarding antioxidant performance should be interpreted cautiously due to possible interference in thiobarbituric acid reactive substances (TBARS) measurements. Sesame oil decreased saturated fatty acids and increased polyunsaturated fatty acids but also increased susceptibility to lipid oxidation. Overall, partial meat replacement, particularly at 50%, demonstrated technological feasibility under the studied conditions; however, nutritional trade‐offs and methodological limitations should be considered when interpreting the results and their applicability.

**Practical Applications:**

This study presents a manufacturing approach for hybrid and plant‐based burgers, using freeze‐dried açaí pulp as an antioxidant and natural colorant and sesame oil as a beef fat substitute. Partial meat substitution (especially up to 50%) improved the technological, morphological, and textural attributes of the formulations. These results can assist food manufacturers in developing products with enhanced nutritional characteristics, using simple, quick processing methods, and incorporating regional raw materials.

## 1 Introduction

According to the World Health Organization (WHO), demand for food is expected to increase from 59% to 98% in 2050 to feed the global population, driven by a population approaching 10 billion (WHO 2021). The demand for animal‐based proteins may double during this period, as meat remains a major source of essential nutrients, including protein, iron, zinc, and vitamins (Jafarzadeh et al. 2024).

However, ethical concerns related to animal welfare, environmental impacts, and health risks associated with excessive meat consumption have intensified debates around reducing animal‐based diets (Xie et al. 2024). Consequently, alternatives such as plant‐based, insect‐based, cell‐based, algae‐derived products, and mycoproteins have gained prominence as more sustainable options (Lima et al. 2023).

Industries have responded by developing products like plant‐based milks, burgers, seaweed bacon, and mushroom‐based items (Peeters et al. 2024). These alternatives aim to mimic the sensory attributes of meat while offering benefits such as cholesterol‐free formulations, lower fat content, and reduced environmental impacts (Seo et al. 2023).

Despite these advantages, plant‐based foods often have lower protein quality and lower levels of essential amino acids, although they provide fiber and bioactive compounds (Wanders et al. 2025). To improve their characteristics, these products frequently incorporate thickeners, colorants, flavorings, and vitamins, raising concerns about ingredient composition, clean‐label trends, and the consumption of ultra‐processed foods (Shireen and Wright 2024).

The transition to vegetarian or vegan diets faces many challenges. As a response, hybrid products combining animal and plant proteins have emerged as an option for flexitarians, offering sensory qualities similar to those of animal proteins with a healthier profile (Li et al. 2025). This search for healthier formulations also includes replacing animal fats, given the risks associated with excessive intake of saturated and trans fats. In this context, sesame oil stands out as a promising substitute due to its high levels of polyunsaturated fatty acids (PUFAs) and bioactive compounds (Feng et al. 2025).

Another challenge for the meat industry is ensuring product stability and preventing oxidation, which traditionally requires synthetic antioxidants such as BHA, BHT, nitrites, and nitrates (Hadidi et al. 2022). Color stability, essential for consumer acceptance, also relies on synthetic dyes (Echegaray et al. 2023). However, concerns about the toxicity of these additives have driven interest in natural alternatives. Spice, fruit, and plant residue extracts have shown antioxidant potential, whereas natural pigments like anthocyanins, carotenoids, chlorophylls, and betalains are being explored as safer colorants (Nabi et al. 2023).

Among these pigments, anthocyanins stand out for their vibrant colors and strong antioxidant, anti‐inflammatory, and anticancer activities (Matuda et al. 2024). The açaí (*Euterpe oleracea* Mart.) is a rich natural source of anthocyanins, making it a promising ingredient for innovative products and a potential natural additive for the food industry (Silveira et al. 2023).

Therefore, this study aimed to develop burgers that partially or completely replace beef with plant‐based ingredients. A simple, quick approach was used rather than ultra‐processing methods. Sesame oil was used as an alternative to beef fat, and freeze‐dried açaí pulp was used as an antioxidant and natural coloring. The impact of the substitutions on the formulations’ physicochemical, chemical, and physical properties was evaluated, as well as their physicochemical and physical stability over 120 days under freezing conditions.

## 2 Materials and Methods

### 2.1 Elaboration of Burger Formulations

The ingredients were purchased at the São Sebastião Market (Fortaleza, CE), except for the freeze‐dried açaí pulp, which was obtained in partnership with Agropar (Paracuru, CE). Ground beef (top round) was packaged in airtight bags and stored in a thermal cooler with ice (4°C ± 1°C) to maintain the cold chain. All ingredients were sent to the Meat and Fish Laboratory (LABCAP) of the Department of Food Engineering (DEAL) of the Federal University of Ceará (UFC) in Fortaleza, CE.

The formulations were defined on the basis of experimental pretests conducted by professors and students with gastronomic experience, aiming to establish viable proportions for the product's technological and structural characteristics. These tests allowed adjusting the levels of meat substitution with vegetable ingredients, ensuring adequate shaping and a visual standard compatible with conventional burgers.

The proportions of rice, textured soy protein, cassava starch, sesame oil, açaí, and mineral water were adjusted in proportion to the increase in beef substitution. The amount of water added (1.31%, 10.87%, 21.74%, 32.61%, and 43.48%) varied across formulations to maintain adequate hydration and product texture. The amount of sesame oil was determined to compensate for the reduction in beef fat. It was progressively adjusted across formulations to maintain a lipid content similar to that of the control product. The concentration of açaí was established based on its ability to impart color and antioxidant potential without compromising the product's technological and visual characteristics. It was therefore kept constant across all formulations. Salt and spices were kept in fixed quantities to standardize the formulation (Table 1).

**TABLE 1:** Burger formulations with partial or total replacement of beef by vegetable ingredients.

Ingredients (%)	100ANI	75ANI25VEG	50ANI50VEG	25ANI75VEG	100VEG
Beef	94.21	70.66	47.11	23.55	—
Risotto rice	—	7.25	14.50	21.75	29.00
Textured soy protein	—	2.72	5.44	8.16	10.88
Cassava starch	—	1.81	3.63	5.44	7.25
Mineral water	1.31	10.87	21.74	32.61	43.48
Sesame oil	—	0.91	1.81	2.72	3.63
Commercial antioxidant	0.14	—	—	—	—
Dehydrated açaí	—	1.46	1.46	1.46	1.46
Salt	1.46	1.46	1.46	1.46	1.46
Garlic powder	0.86	0.86	0.86	0.86	0.86
Onion powder	0.86	0.86	0.86	0.86	0.86
Smoked paprika powder	0.73	0.73	0.73	0.73	0.73
Black pepper powder	0.21	0.21	0.21	0.21	0.21
Parsley powder	0.21	0.21	0.21	0.21	0.21
**Total**	**100.0**	**100.0**	**100.0**	**100.0**	**100.0**

*Note*: 100ANI: 100% animal burger; 75ANI25VEG: 75% animal/25% vegetable burger; 50ANI50VEG: 50% animal/50% vegetable burger; 25ANI75VEG: 25% animal/75% vegetable burger; 100VEG: 100% vegetable burger. *n* = 3 repetitions. Commercial antioxidant: ascorbic acid.

Five burger formulations were developed by replacing beef with vegetable ingredients at 0% (100ANI), 25% (75ANI25VEG), 50% (50ANI50VEG), 75% (25ANI75VEG), and 100% (100VEG), following the methodology of Janardhanan et al. (2023) with adaptations. The adaptations included the use of risotto rice powder, sesame oil as a fat replacer, freeze‐dried açaí pulp as an antioxidant/colorant, and adjustments to water levels based on the degree of meat replacement. The vegetable base consists of all ingredients except beef and commercial antioxidants.

Ten burgers of each of the five formulations were produced in three independent replicates (batches) using the same manufacturing approach. The ingredients were weighed on a semi‐analytical balance (TS2KS model, OHAUS, Parsippany, NJ, USA).

Risotto rice and textured soy protein were crushed in a household blender (Turbo Chef 7‐in‐1 model, MONDIAL Ltd., São Paulo, SP, Brazil) until a fine powder was obtained.

Risotto rice was chosen because it produces a finer, more homogeneous powder than traditional rice. The Carnaroli variety was selected for its higher starch content, which can enhance the product's technological properties.

In the 100ANI burger, dry ingredients were mixed with water and incorporated into the beef. In the hybrid formulations, powdered ingredients were first hydrated and combined with sesame oil to form the vegetable base, and then beef was added. The 100VEG formulation followed the same procedure but without incorporating beef.

The burgers were shaped using a manual shaping machine (model 230500K, Kikas Máquinas Ltd., São Paulo, SP, Brazil), with an average weight of 90.0 g and a thickness of 2.0 cm. They were packed in Styrofoam trays, wrapped in polyvinyl chloride (PVC) plastic film, a stretchable type with an approximate thickness of 9 µm, commonly used for packaging fresh foods, and with a gas‐permeability characteristic of this material. Then they were stored frozen (−18°C ± 1°C) until analysis, conducted the same week as processing, without compromising their quality characteristics.

### 2.2 Characterization of Burger Formulations

Initially, the burgers were thawed (4°C ± 1°C/24 h). For the raw burger analyses, the samples were ground to ensure greater homogeneity and representativeness. For post‐cooking evaluations, the patties were fried on a gas griddle heated to 150°C ± 1°C with soybean oil until the center reached 75°C ± 1°C (measured with an infrared thermometer) and were turned every 2 min until they presented a characteristic burger appearance. The analyses described below were performed in triplicate after cooling to room temperature (25°C ± 1°C).

#### 2.2.1 pH, Water Activity, and Lipid Oxidation

pH, water activity, and lipid oxidation were measured on the raw samples. The burgers’ pH was measured using a digital pH meter (model Tec‐5, Tecnal, Piracicaba, SP, Brazil) (method 981.12, Association of Official Agricultural Chemists [AOAC] 2023). The water activity (*a*_w_) was determined using a water activity meter (model 4TE, Aqualab, Pullman, United States [method 978.18]; AOAC 2023). Lipid oxidation was determined by the thiobarbituric acid reactive substances (TBARS) method (Raharjo et al. 1992; Facco 2002), with readings at 531 nm using a spectrophotometer (model SP‐22, Biospectro Ltd., Curitiba, PR, Brazil). No specific correction for potential interference from sample pigments was applied. Results were expressed as mg MDA kg^−1^ of sample.

#### 2.2.2 Centesimal Composition and Energy Value

The centesimal composition assessment was performed on the raw products (AOAC 2023). Moisture was measured using the gravimetric method, on the basis of the determination of the weight loss of the sample subjected to heating in an oven at 105°C (950.46); protein content was determined by the Kjeldahl method (960.52); ash content by incineration at 550°C (920.153); and lipid content by the Soxhlet extraction method (991.36).

For protein content calculation, a factor of 6.25 (for meat proteins or protein mixtures) was used for both 100% animal and hybrid formulations. For the 100% vegetable formulation, the factor 5.75 (other vegetable proteins) was used (Brazil 2020). The carbohydrate was calculated by difference, and the energy value using Atwater's conversion factors (4 kcal g^−1^ for protein and carbohydrates, 9 kcal g^−1^ for lipids) (Osborne and Voogt 1978).

#### 2.2.3 Total Phenolic Compounds and Total Antioxidant Activity

Extracts were prepared from crude samples, following the method of Larrauri et al. (1997). They were obtained and stored in the dark, given that many phenolics are photosensitive. Approximately 1.0 g of the sample was weighed, and two successive extractions were performed with 50% methanol and 70% acetone. After resting, the samples were centrifuged, and the supernatants were filtered and collected in a 50 mL volumetric flask, completing the volume with distilled water. Finally, the extracts were filtered again to remove suspended particles.

The analysis of total phenolics followed the methodology of Obanda et al. (1997). The calibration curve was obtained from the gallic acid stock solution (50 mg L^−1^). Then, a 400.0 µL aliquot of the extract and 100.0 µL of distilled water were used. The tubes were shaken and left to stand for 30 min. After this time, the reading was taken in a spectrophotometer at 700 nm, and the results were expressed as mg GAE 100 g^−1^. For hybrid and plant‐based formulations, readings were taken after 4 h, as turbidity developed upon addition of sodium carbonate.

The ABTS^•+^ (2,2′‐Azino‐bis(3‐ethylbenzthiazoline‐6‐sulfonic acid)) free radical scavenging capacity assay followed the methodology of Re et al. (1999). The calibration curve was obtained from the Trolox stock solution (2000 µM). Then, three different aliquots of the extract were added to test tubes: 26.0, 28.0, and 30.0 µL of the extract, with 4.0, 2.0, and 0.0 µL of distilled water, respectively. The tubes were shaken and left to stand for 6 min. After this time, the reading was taken in a spectrophotometer at 734 nm, with results expressed in µM Trolox g^−1^.

The ferric reducing antioxidant power (FRAP) assay followed the method of Benzie and Strain (1996). The calibration curve was obtained from a stock solution of ferrous sulfate (FeSO_4_, 2000 µM). Then, three different aliquots of the extract were added to test tubes: 70.0, 80.0, and 90.0 µL, with 20.0, 10.0, and 0.0 µL of distilled water, respectively. The tubes were shaken and left to stand for 30 min. After this period, the reading was taken in a spectrophotometer at 595 nm, and the results were expressed in µM FeSO_4_ g^−1^.

#### 2.2.4 Fatty Acid Profile

The lipid fraction of the raw samples was extracted cold using the Bligh and Dyer (1959) method. Fatty acid methyl esters were prepared according to the 055/IV methods (IAL 2008) and 996.01 (AOAC 2023). Approximately 0.10 g of the samples were weighed, and 3.0 mL of *n*‐hexane and 0.2 mL of 2 M methanolic potassium hydroxide solution were added. After vortexing, 3.0 mL of saturated sodium chloride solution was added, and the vials were left to stand until phase separation occurred. The top layer was recovered for chromatographic analysis.

Quantification (053/IV; IAL 2008) was performed in a gas chromatograph with flame ionization detector (model FOCUS, Thermo Scientific Inc., MA, United States), equipped with a capillary column (model SP‐2560, Supelco, Darmstadt, Germany, 100 m long × 0.25 mm internal diameter × 0.20 µm film thickness), under the following conditions—injection mode: split (ratio 1:10); sample injection volume: 1.0 µL; detector temperature: 260°C ± 1°C; injector temperature: 250°C ± 1°C; temperature programming: starting at 100°C ± 1°C, with a rate of 4°C min^−1^ until reaching 220°C ± 1°C, maintaining for 10 min, then with a rate of 4°C min^−1^ until reaching 250°C ± 1°C, for 7 min; carrier gas: nitrogen; flow rate: 1.0 mL min^−1^.

The conversion factors were calculated from chromatograms obtained using the standard sample (FAME Mix 37, Supelco, Darmstadt, Germany). The result was expressed in g 100 g^−1^ of fat.

#### 2.2.5 Metabolic Profile

Polar metabolites were extracted from the raw samples following the protocol of Lisec et al. (2006). The samples were macerated in liquid nitrogen until a homogeneous powder was obtained. Subsequently, the polar metabolites were extracted with methanol (700.0 µL) and ribitol (30.0 µL), followed by agitation, centrifugation, and phase separation with chloroform (375.0 µL) and ultrapure water (750.0 µL). The polar phase was collected, dried in a vacuum concentrator, and subjected to derivatization with methoxyamine hydrochloride in pyridine (20.0 µL) and MSTFA (*N*‐Methyl‐*N*‐trimethylsilyl‐trifluoroacetamide) (35.0 µL) under controlled agitation.

For the quantitative determination of the metabolites, a gas chromatograph coupled to mass spectrometry with a quadrupole‐type analyzer was used (model GC‐EI‐Q‐MS QP‐PLUS 2010, Shimadzu Ltd., Kyoto, Japan), equipped with a capillary column (model SPB‐5, Supelco, Darmstadt, Germany, 30 m long × 0.25 mm internal diameter × 0.25 µm film thickness), under the following conditions—injection mode: split (ratio 1:10); sample injection volume: 1.0 µL; detector temperature: 250°C ± 1°C; injector temperature: 230°C ± 1°C; temperature of the 70 eV electric impact ion source: 200°C ± 1°C; transfer line (interface) temperature: 300°C ± 1°C; total ion counting mode: 80–700 *m*/*z*; temperature programming: start at 80°C ± 1°C, maintain for 5 min, then increase at a rate of 10°C min^−1^ until reaching 310°C ± 1°C; carrier gas: helium; flow rate: 1.0 mL min^−1^.

The metabolic profile was obtained from the analysis of chromatograms and mass spectra using the software Xcalibur 2.1 (Thermo Scientific Inc., Massachusetts, United States) and the Golm Metabolome database (Kopka et al. 2005). The results were reported as relative metabolic content (RMC), identifying amino acids, organic acids, sugars, and polyalcohols. The result was expressed in g^−1^.

#### 2.2.6 Appearance and Instrumental Color Parameters

The images were obtained under similar lighting conditions, and instrumental measurements were performed at three positions on each side of the burger. The digital images were captured, and the color parameters were evaluated on both raw and fried burgers.

Digital images were captured in JPEG format (Joint Photographic Experts Group) using a digital camera (model Galaxy M54, 108MP, f/1.8 aperture, Samsung Ltd., Manaus, AM, Brazil).

Instrumental color parameters (lightness—L*, red–green axis—a*, yellow–blue axis—b*, color intensity—C*, and hue angle—*H*°) were measured in the CIELAB space with a portable digital colorimeter (model NR60CP+, Shenzhen ThreeNH Technology Co. Ltd., Shenzhen, China), with a D_65_ illuminant and 10° standard observer (McGuire 1992).

#### 2.2.7 Cooking Loss, Shrinkage, and Yield

Cooking loss was evaluated (Equation 1) as described by the American Meat Science Association (AMSA 2016); shrinkage (Equation 2) and yield (Equation 3) were evaluated according to Berry (1992). After frying, excess oil was removed from the burgers’ surfaces with absorbent paper before weighing to minimize the effect of oil absorption on the results. The results were expressed in %.

(1) $$\mathrm{Cooking}\;\mathrm{loss}=\frac{\mathrm{raw}\;\mathrm{sample}\;\mathrm{mass}\left(g\right)-\;\mathrm{fried}\;\mathrm{sample}\;\mathrm{mass}\left(g\right)}{\mathrm{raw}\;\mathrm{sample}\;\mathrm{mass}\left(g\right)}\times 100$$

(2) $$\mathrm{Shrinkage}=\frac{\mathrm{diameter}\;\mathrm{of}\;\mathrm{raw}\;\mathrm{sample}\left(\mathrm{mm}\right)-\;\mathrm{diameter}\;\mathrm{of}\;\mathrm{the}\;\mathrm{fried}\;\mathrm{sample}\left(\mathrm{mm}\right)}{\mathrm{diameter}\;\mathrm{of}\;\mathrm{raw}\;\mathrm{sample}\left(\mathrm{mm}\right)}\times 100$$

(3) $$\mathrm{Yield}=\frac{\mathrm{fried}\;\mathrm{sample}\;\mathrm{mass}\left(g\right)}{\mathrm{raw}\;\mathrm{sample}\;\mathrm{mass}\left(g\right)}\times 100$$

#### 2.2.8 Microstructure

Raw samples were prepared following the method of Nascimento et al. (2024), with adaptations. The burgers (1.0 cm long, 1.0 cm wide, and 0.5 cm high) were fixed in a compound solution (2.5% glutaraldehyde (v/v), 4.0% formaldehyde, and 0.2 M phosphate buffer (pH 7.2) in equal proportions) for 24 h at 4°C ± 1°C, washed three times with the same buffer (10 min each), and dehydrated in an acetone series (30%, 60%, 90%, and 100%), 20 min each. The 100% vegetable formulation disintegrated upon contact with the compound solution.

It was subjected to dehydration in an oven with air circulation (model SSDc 30 L, SolidSteel Ltd., São Paulo, SP, Brazil) at 50°C ± 1°C for 2 h. The samples were mounted on a metal support (stub) with carbon tape and coated with 20 nm of gold by spray coating in a metalizing machine (model QT150 ES, Quorum, Edinburgh, United Kingdom). Morphology was observed under a scanning electron microscope (model Quanta 450 FEG, MEV‐FEI, Oregon, United States) at 2000× magnification and 20 kV acceleration voltage.

#### 2.2.9 Texture Profile

The evaluation of the texture profile analysis (TPA) of the fried formulations was performed in a texture analyzer (model TA‐TX2i, Stable Micro Systems Ltd., Surrey, United Kingdom), evaluating hardness (N), elasticity (mm), cohesiveness, gumminess (N), and chewiness (N mm) (Bourne 1978). The burgers were cut with a stainless‐steel blade for cutting food samples, obtaining 1.6 cm in diameter and 2.0 cm in height.

The TPA used a P35 probe, equipped with a 30 kg load cell, test speed was 1 mm s^−1^, with a trigger of 5 g. The samples were subjected to two consecutive cycles of 50% compression at room temperature (25°C ± 1°C), with an average of 10 replicates per cycle.

#### 2.2.10 Physicochemical and Physical Stability

The stability of the burgers under freezing (−18°C ± 1°C) was evaluated every 30 days, for a period of 4 months: 24 h (T0); 30 days (T30); 60 days (T60); 90 days (T90); and 120 days (T120) after preparation. pH, lipid oxidation (TBARS), cooking loss, shrinkage, and yield were evaluated.

### 2.3 Statistical Analysis

For each batch, three burgers were randomly selected and analyzed in triplicate. The batches were considered independent biological replicates. The experiment was conducted using a split‐plot factorial design with five formulations in the main plots, 5 days of storage in the subplots, and three replications. The effects of interactions (independence) between these factors were evaluated. The software used was the XLSTAT version 2024.4.2.1426 (Lumivero, LLC, Paris, France).

The results obtained were expressed as the mean and standard deviation. The data from the formulation characterization were analyzed using analysis of variance (ANOVA), followed by Tukey's test and Bonferroni's correction at the 5% significance level. The stability results were subjected to ANOVA, Type I, II, and III sum‐of‐squares analyses, regression analysis, and a paired multiple‐comparisons test using Tukey's test.

## 3 Results and Discussion

### 3.1 pH, Water Activity, and Lipid Oxidation

Increasing the substitution of beef with vegetable ingredients raised the pH and lipid oxidation of the burgers, while reducing their water activity (Table 2).

**TABLE 2:** Values of pH, water activity, and lipid oxidation of burger formulations with partial or total replacement of beef by vegetable ingredients (mean ± SD).

Formulations	Parameters		
	pH	Water activity	Lipid oxidation (mg MDA kg^−1^)
**100ANI**	5.65 ± 0.01^d^	0.978 ± 0.00^a^	0.86 ± 0.03^e^
**75ANI25VEG**	5.77 ± 0.01^c^	0.977 ± 0.00^a^	1.32 ± 0.05^d^
**50ANI50VEG**	5.84 ± 0.09^c^	0.976 ± 0.00^a^	1.39 ± 0.03^c^
**25ANI75VEG**	6.26 ± 0.01^b^	0.974 ± 0.00^b^	1.57 ± 0.03^b^
**100VEG**	6.44 ± 0.01^a^	0.973 ± 0.00^b^	2.43 ± 0.04^a^

*Note*: 100ANI: 100% animal burger; 75ANI25VEG: 75% animal/25% vegetable burger; 50ANI50VEG: 50% animal/50% vegetable burger; 25ANI75VEG: 25% animal/75% vegetable burger; 100VEG: 100% vegetable burger. Means followed by the same letters in the same column do not differ from each other by Tukey's test (*p* ≥ 0.05). *n* = 3 repetitions.

Abbreviation: MDA, malondialdehyde.

The samples differed significantly in pH (*p* ≤ 0.05), except for 75ANI25VEG and 50ANI50VEG. The lowest pH was observed in 100ANI, with a progressive increase in the hybrid formulations, and the highest value was observed in 100VEG (Table 2).

This behavior can be attributed to the protein content of meat and the differences in the composition and structure of plant proteins, such that replacing meat with plant‐based ingredients increases pH due to the greater alkalinity and the diversity of components used in the formulations (De Marchi et al. 2021). Janardhanan et al. (2023) observed similar pH levels in meat, plant‐based (soy, rice, and beans), and hybrid burgers.

Water activity (*a*_w_) differed among samples: 25ANI75VEG and 100VEG were similar to each other (*p* ≥ 0.05) but differed from the others (*p* ≤ 0.05). Higher *a*_w_ was observed in formulations with more meat, whereas vegetable‐rich samples showed lower aw values (Table 2). Flores et al. (2025) produced pork burgers and hybrids with 38% replacement of pork with textured pea protein, with an average aw of 0.970 across all samples, similar to the present study.

Regarding lipid oxidation, all samples differed significantly from each other (*p* ≤ 0.05) (Table 2). The freeze‐dried açaí pulp was used as a dye and antioxidant, replacing ascorbic acid. Higher average malondialdehyde values were observed in hybrid and plant‐based samples. This behavior may be related to the increased levels of PUFAs resulting from the use of sesame oil as a substitute for beef fat in these formulations. Given that PUFAs are more susceptible to oxidation due to their multiple double bonds, the formation of various oxidative metabolites and reactive oxygen species is favored, intensifying the oxidative process (Islam et al. 2023).

In this context, the meat raw material used to prepare the hamburgers was also evaluated, with a low lipid oxidation value (0.17 ± 0.01 mg MDA kg^−1^), demonstrating adequate integrity and quality for processing. Similarly, the 100ANI formulation without freeze‐dried açaí pulp showed a lower TBARS value (0.86 ± 0.03 mg MDA kg^−1^).

The results suggest that some of the values observed in formulations containing açaí may be due to interference from anthocyanins in the matrix, affecting the spectrophotometric readings at 531 nm and potentially leading to an overestimation of malondialdehyde by the TBARS method (Bellucci et al. 2022). Therefore, the results must be interpreted with caution, considering that the lack of blank correction or complementary analytical methods represents an important limitation of the study.

Therefore, although all formulations presented TBARS values below the threshold of 2.5 mg MDA kg^−1^ reported for meat products (Domínguez et al. 2019), this result cannot be interpreted as definitive evidence of oxidative stability. Similarly, the potential antioxidant effect of freeze‐dried açaí pulp should be interpreted cautiously under these experimental conditions.

Due to the methodological limitations associated with anthocyanin interference, TBARS results should be interpreted only as indicative estimates rather than definitive measures of lipid oxidation.

Oliveira et al. (2022) replaced ascorbic acid and sodium erythorbate with encapsulated olive leaf extract in sheep beef burgers, showing, by TBARS analysis, lower antioxidant efficacy of the extract in relation to vitamin C, due to the latter's superior ability to inactivate free radicals during the initiation and propagation phases of lipid oxidation.

Future studies should incorporate complementary methods for lipid oxidation assessment, such as peroxide value, hexanal quantification, and conjugated dienes, along with appropriate blank corrections, to provide a more robust evaluation of oxidative stability in formulations containing pigmented ingredients. The following section presents the centesimal composition and energy value of the formulations, providing further insight into the impact of meat replacement on their nutritional characteristics.

### 3.2 Centesimal Composition and Energy Value

In the moisture analysis, all samples differed significantly from each other (*p* ≤ 0.05), and as expected, replacing meat with plant‐based ingredients reduced the water content of the formulations (Table 3). Janardhanan et al. (2023) observed a similar pattern in their study, with the animal‐origin sample recording the highest moisture content (75.30%), followed by hybrids (65.01%–65.95%) and vegetables (56.42%).

**TABLE 3:** Proximate composition and energy value of burger formulations with partial or total replacement of beef by vegetable ingredients (mean ± SD).

Formulations	Parameters					
	Moisture (%)	Ashes (%)	Lipids (%)	Protein (%)	Carbohydrates (%)	Energy value (kcal 100 g^−1^)
**100ANI**	71.93 ± 1.28^a^	2.68 ± 0.18^b^	3.57 ± 1.07^ab^	19.54 ± 0.37^a^	2.29 ± 0.51^e^	119.44 ± 10.65^d^
**75ANI25VEG**	64.06 ± 0.54^b^	2.75 ± 0.03^b^	3.67 ± 0.48^ab^	15.89 ± 0.30^b^	13.64 ± 0.32^d^	151.11 ± 4.36^c^
**50ANI50VEG**	58.88 ± 0.48^c^	2.68 ± 0.05^b^	3.88 ± 0.33^ab^	12.03 ± 0.23^c^	22.53 ± 0.44^c^	173.14 ± 2.88^b^
**25ANI75VEG**	50.63 ± 1.20^d^	3.22 ± 0.13^a^	4.50 ± 0.40^a^	10.20 ± 0.24^d^	31.46 ± 1.17^b^	207.08 ± 5.99^a^
**100VEG**	46.81 ± 0.68^e^	3.18 ± 0.19^a^	3.23 ± 0.70^b^	7.43 ± 0.31^e^	39.36 ± 0.98^a^	216.18 ± 2.78^a^

*Note*: 100ANI: 100% animal burger; 75ANI25VEG: 75% animal/25% vegetable burger; 50ANI50VEG: 50% animal/50% vegetable burger; 25ANI75VEG: 25% animal/75% vegetable burger; 100VEG: 100% vegetable burger. *n* = 3 repetitions. Means followed by the same letters in the same column do not differ from each other by Tukey's test (*p* ≥ 0.05).

Replacing up to 50% of beef with vegetables did not affect ash content, as 100ANI, 75ANI25VEG, and 50ANI50VEG samples were similar (*p* ≥ 0.05) but differed from 25ANI75VEG and 100VEG (*p* ≤ 0.05), which were also similar to each other (*p* ≥ 0.05). The highest ash content was observed in the 25ANI75VEG formulation, followed by the 100VEG sample and the others (Table 3). Penalver et al. (2025) obtained a similar behavior, recording 2.39% for the control sample, 3.72% for the hybrid formulation, and 3.16% for the plant‐based burger.

Lipid content was similar among samples (*p* ≥ 0.05), except for 25ANI75VEG and 100VEG, which differed significantly (*p* ≤ 0.05) (Table 3). The Ministry of Agriculture and Livestock (Ministério da Agricultura e Pecuária—MAPA), through Ordinance No. 724, of December 23, 2022, approved the Technical Regulation of Identity and Quality of the burger, which sets a maximum fat content of 25%, and all formulations, including plant‐based ones, complied with this limit (Brazil 2022).

Fat is highly relevant in meat products, contributing to flavor, juiciness, and texture. However, excessive consumption is associated with an increased risk of cardiovascular disease and obesity (Lee et al. 2023). Therefore, we opted for low‐fat meat, without adding extra fat beyond that naturally present in the raw material.

Sesame oil was used as a substitute for beef fat in hybrid burgers and vegetable burgers due to its functional components (lignans, vitamin E, carotenoids, coenzyme Q, and tocotrienols) and its pleasant aroma and taste (Feng et al. 2025). This substitution proved effective, as lipid content was similar except in the formulations 25ANI75VEG and 100VEG. Flores et al. (2025) replaced animal fat with coconut and sunflower oils in hybrid burgers and compared them with the control (pork) but did not observe a significant difference in fat content, which ranged from 17.3% to 19.0%.

Protein content differed significantly among all samples (*p* ≤ 0.05), with a reduction proportional to the substitution of meat (Table 3). The minimum protein content in burgers is 15% (Brazil 2022). Besides the control (100ANI), only the 75ANI25VEG formulation met the minimum required limit, and there is currently no specific legislation in Brazil for hybrid or plant‐based analogous products. Penalver et al. (2025) obtained about 17% in the animal sample, 15% in the hybrid sample, and 12% in the vegetable sample.

This reduction may also reflect lower protein quality, as plant‐based proteins generally have a less balanced essential amino acid profile than animal proteins. Therefore, depending on the formulation, protein complementation strategies may be required to ensure adequate nutritional value (Wanders et al. 2025).

Carbohydrate content differed among all samples (*p* ≤ 0.05) and increased with meat replacement by vegetables (Table 3). The legislation establishes a maximum of 3% total carbohydrates for burgers (Brazil 2022). Thus, the 100ANI formulation meets the requirement, whereas the others cannot be evaluated using this parameter, as they have different compositions from those provided for burgers.

In the absence of specific legislation for hybrid and analogous plant‐based products in Brazil, these formulations should not be evaluated on the basis of the limits set for conventional meat burgers; it is more appropriate to compare them with similar products described in the literature.

In this context, carbohydrate content can influence consumer acceptance, as it is related to perceptions of healthiness, energy value, and adequacy for specific diets; however, in plant products or hybrids, higher values are expected due to the incorporation of plant‐based ingredients. They should not be interpreted in isolation as negative factors, but rather in conjunction with expectations for the product category.

Regarding the energy value, the samples differed significantly from each other (*p* ≤ 0.05), except for 25ANI75VEG and 100VEG (*p* ≥ 0.05) (Table 3). The determination of protein, carbohydrate, and lipid contents allowed the calculation of the energy value; higher values were recorded in formulations with greater replacement of meat by vegetable ingredients, a trend similar to that observed for carbohydrate content.

This increase in energy value highlights a nutritional trade‐off, where improvements in lipid profile, due to the use of vegetable oils, may be accompanied by higher caloric density and reduced protein quality (Garnås 2025).

Costa‐Catala et al. (2023) compared the labeling of meat products and their analogues marketed in Spain. They found that vegetable burgers had a higher carbohydrate content, whereas conventional burgers had a higher energy value.

Mercês et al. (2025) developed burgers from green banana biomass derived from teff (a crop native to Ethiopia and Eritrea) and chickpeas and compared them with industrialized samples (animal and vegetable). The meat product contained 3.70% carbohydrates and 285.30 kcal·100 g^−1^, whereas vegetables had between 12.59% and 28.55% of carbohydrates, with energy values ranging from 161.80 to 254.63 kcal·100 g^−1^.

The variations observed in the centesimal composition within the same study are mainly due to differences in the ingredients used in the formulations. When comparing research, such as the present study and the aforementioned works, other factors can also influence results, including the analytical methods employed, the processing techniques adopted, and the storage conditions of the products (de Melo et al. 2024).

The hybrid and plant‐based formulations showed lower average protein content and higher carbohydrate content than the animal‐based burger. Regarding protein content, the results were expected, as meat is a primary source and vegetables are a secondary source. The higher carbohydrate content may suggest the presence of dietary fiber from the plant‐based ingredients used (Berisha et al. 2024). The following section evaluates phenolic compounds and total antioxidant activity, providing further insight into the formulations’ functional properties.

### 3.3 Phenolic Compounds and Total Antioxidant Activity

Regarding phenolic content, samples 50ANI50VEG, 25ANI75VEG, and 100VEG showed similarity to each other (*p* ≥ 0.05), whereas 100ANI and 75ANI25VEG differed from each other and from the other formulations (*p* ≤ 0.05). It can be inferred that the replacement of meat with vegetables between 50% and 100% did not significantly influence the content of phenolics (Table 4).

**TABLE 4:** Total phenolic content (TPC) and total antioxidant activity (2,2′‐Azino‐bis(3‐ethylbenzthiazoline‐6‐sulfonic acid [ABTS] and ferric reducing antioxidant power [FRAP]) of burger formulations with partial or total replacement of beef by vegetable ingredients (mean ± SD).

Formulations	Parameters		
	TPC (mg GAE 100 g^−1^)	ABTS^•+^ (µM Trolox g^−1^)	FRAP (µM FeSO_4_ g^−1^)
**100ANI**	110.74 ± 1.13^a^	13.51 ± 0.70^a^	13.75 ± 0.67^a^
**75ANI25VEG**	51.61 ± 3.42^c^	10.62 ± 0.82^b^	3.63 ± 0.48^c^
**50ANI50VEG**	56.20 ± 2.01^b^	12.26 ± 0.99^ab^	3.29 ± 0.17^c^
**25ANI75VEG**	58.82 ± 1.96^b^	11.15 ± 0.93^b^	5.65 ± 0.26^b^
**100VEG**	56.26 ± 3.23^b^	12.91 ± 1.13^a^	3.38 ± 0.09^c^

*Note*: 100ANI: 100% animal burger; 75ANI25VEG: 75% animal/25% vegetable burger; 50ANI50VEG: 50% animal/50% vegetable burger; 25ANI75VEG: 25% animal/75% vegetable burger; 100VEG: 100% vegetable burger. Means followed by the same letters in the same column do not differ from each other by Tukey's test (*p* ≥ 0.05). *n* = 3 repetitions.

Abbreviations: FeSO_4_, ferrous sulfate; GAE, gallic acid equivalent.

By the ABTS method, 100ANI, 50ANI50VEG, and 100VEG were similar, as were the hybrid formulations (*p* ≥ 0.05), and antioxidant capacities were close across all samples. By the FRAP method, 75ANI25VEG, 50ANI50VEG, and 100VEG were similar (*p* ≥ 0.05), whereas 25ANI75VEG and 100ANI differed from each other and from the others (*p* ≤ 0.05) (Table 4).

Although freeze‐dried açaí pulp is a source of phenolic compounds with recognized antioxidant activity (Carvalho et al. 2025), the results obtained did not demonstrate a direct relationship between the total phenolic content and the antioxidant activity of the formulations. For example, the 50ANI50VEG, 25ANI75VEG, and 100VEG formulations showed similar phenolic contents but different behaviors in the ABTS and FRAP tests. Furthermore, the 100ANI formulation showed greater antioxidant activity in both methods, especially in the FRAP assay, even without a proportional increase in phenolic compounds compared with the other formulations (Table 4).

These results suggest that the observed antioxidant activity does not depend exclusively on phenolic compounds from açaí but also on the contribution of other bioactive compounds present in the meat matrix, such as carnosine, anserine, taurine, glutathione, and coenzyme Q10 (di Corcia et al. 2022; Jairath et al. 2024). Additionally, differences between the ABTS and FRAP methods indicate that different antioxidant mechanisms may have predominated across the formulations, as each assay has specific sensitivities to certain compounds and mechanisms of action (Abdullah et al. 2022).

Therefore, although freeze‐dried açaí pulp has potential as a natural antioxidant, its incorporation into formulations did not consistently increase antioxidant activity, indicating the need to investigate other application strategies and its interactions with the food matrix.

Nascimento et al. (2024) added powdered vinegar leaves (4 and 8 g kg^−1^) as an alternative natural antioxidant in beef burgers. They reported similar behavior, and the formulation containing sodium erythorbate (0.08 g) showed higher phenolic content and antioxidant potential (ABTS^•+^), results similar to those observed in this study.

The following section examines the fatty acid profile of the formulations, providing further information on the impact of meat substitution on lipid composition and its implications for nutritional and technological characteristics.

### 3.4 Fatty Acid Profile

The use of sesame oil as a fat substitute significantly altered the fatty acid profile of the burgers, reducing saturated fatty acids and increasing PUFAs (Table 5), an effect attributed to the high unsaturated fat content in its matrix (Feng et al. 2025).

**TABLE 5:** Fatty acid profile of burger formulations with partial or total replacement of beef by vegetable ingredients (mean ± SD).

Fatty acids (g 100 g^−1^ of fat)	Formulations				
	100ANI	75ANI25VEG	50ANI50VEG	25ANI75VEG	100VEG
**C14:0**	0.10 ± 0.04^a^	0.07 ± 0.01^ab^	0.05 ± 0.01^bc^	0.03 ± 0.00^cd^	0.01 ± 0.00^d^
**C15:0**	0.03 ± 0.02^a^	0.01 ± 0.00^ab^	0.01 ± 0.00^b^	0.01 ± 0.01^b^	0.00 ± 0.00^b^
**C16:0**	0.88 ± 0.25^a^	0.78 ± 0.09^a^	0.72 ± 0.13^a^	0.69 ± 0.09^a^	0.42 ± 0.05^b^
**C17:0**	0.04 ± 0.02^a^	0.03 ± 0.01^ab^	0.02 ± 0.01^bc^	0.01 ± 0.00^cd^	0.00 ± 0.00^d^
**C18:0**	0.58 ± 0.19^a^	0.44 ± 0.06^ab^	0.34 ± 0.03^bc^	0.26 ± 0.02^cd^	0.10 ± 0.02^d^
**C20:0**	0.02 ± 0.02^a^	0.04 ± 0.03^a^	0.07 ± 0.04^a^	0.09 ± 0.06^a^	0.08 ± 0.06^a^
**C21:0**	0.01 ± 0.01^a^	0.01 ± 0.01^a^	0.00 ± 0.01^a^	0.01 ± 0.01^a^	0.00 ± 0.00^a^
**C22:0**	0.00 ± 0.00^a^	0.01 ± 0.01^a^	0.01 ± 0.01^a^	0.01 ± 0.01^a^	0.01 ± 0.01^a^
**C23:0**	0.01 ± 0.01^a^	0.01 ± 0.01^a^	0.01 ± 0.01^a^	0.01 ± 0.00^a^	0.00 ± 0.00^a^
***Saturated***	1.67 ± 0.50^a^	1.40 ± 0.14^ab^	1.23 ± 0.15^b^	1.12 ± 0.10^b^	0.62 ± 0.06^c^
**C14:1**	0.02 ± 0.01^a^	0.01 ± 0.00^ab^	0.01 ± 0.00^b^	0.01 ± 0.01^c^	0.00 ± 0.00^c^
**C16:1**	0.14 ± 0.04^a^	0.10 ± 0.02^ab^	0.08 ± 0.01^bc^	0.05 ± 0.00^cd^	0.01 ± 0.01^d^
**C17:1**	0.03 ± 0.01^a^	0.02 ± 0.00^b^	0.01 ± 0.00^c^	0.01 ± 0.01^cd^	0.00 ± 0.00^d^
**C18:1n9c (*ω*** −** 9)**	1.30 ± 0.35^a^	1.35 ± 0.15^a^	1.33 ± 0.08^a^	1.38 ± 0.15^a^	0.88 ± 0.15^b^
**C18:1n9t (*ω*** −** 9)**	0.10 ± 0.04^a^	0.07 ± 0.01^b^	0.05 ± 0.01^bc^	0.03 ± 0.01^cd^	0.00 ± 0.00^d^
**C20:1n9 (*ω*** −** 9)**	0.00±0.00^b^	0.01 ± 0.01^a^	0.00 ± 0.00^b^	0.01 ± 0.00^a^	0.01 ± 0.01^ab^
***Monounsaturated***	1.59 ± 0.44^a^	1.56 ± 0.17^a^	1.48 ± 0.10^a^	1.48 ± 0.16^a^	0.89 ± 0.16^b^
**C18:2n6c (*ω*** −** 6)**	0.11 ± 0.05^d^	0.52 ± 0.09^c^	0.99 ± 0.05^b^	1.53 ± 0.22^a^	1.30 ± 0.24^a^
**C20:3n6 (*ω*** −** 6)**	0.01 ± 0.01^a^	0.01 ± 0.01^ab^	0.00 ± 0.01^b^	0.00 ± 0.01^b^	0.00 ± 0.01^b^
**C20:4n6 (*ω*** −** 6)**	0.01 ± 0.01^a^	0.01 ± 0.01^a^	0.01 ± 0.01^a^	0.01 ± 0.01^a^	0.01 ± 0.01^a^
**C18:3n3 (*ω*** −** 3)**	0.01 ± 0.02^a^	0.02 ± 0.03^a^	0.03 ± 0.05^a^	0.05 ± 0.08^a^	0.04 ± 0.06^a^
**C20:3n3 (*ω*** −** 3)**	0.02 ± 0.02^a^	0.01 ± 0.01^ab^	0.01 ± 0.01^ab^	0.00 ± 0.01^b^	0.00 ± 0.01^b^
**C20:5n3 (*ω*** −** 3)**	0.01 ± 0.01^a^	0.01 ± 0.01^ab^	0.01 ± 0.01^ab^	0.00 ± 0.00^b^	0.00 ± 0.00^b^
***Polyunsaturated***	0.17 ± 0.07^d^	0.58 ± 0.10^c^	1.05 ± 0.05^b^	1.59 ± 0.23^a^	1.35 ± 0.26^a^

*Note*: 100ANI: 100% animal burger; 75ANI25VEG: 75% animal/25% vegetable burger; 50ANI50VEG: 50% animal/50% vegetable burger; 25ANI75VEG: 25% animal/75% vegetable burger; 100VEG: 100% vegetable burger. *n* = 3 repetitions. Means followed by the same letters in the same line do not differ from each other by Tukey's test (*p* ≥ 0.05).

For saturated fatty acids, the 100ANI and 75ANI25VEG samples were statistically similar, as were the other hybrid formulations (*p* ≥ 0.05), whereas 100VEG differed from all the others (*p* ≤ 0.05) (Table 5). The higher levels of myristic (C14:0), palmitic (C16:0), and stearic (C18:0) acids in beef burgers are related to their presence in animal adipose tissue, a significant endocrine organ and energy reservoir (Nawaz et al. 2024). Oh et al. (2025) also reported higher concentrations of saturated fatty acids in meat products than in plant analogues.

Among the monounsaturated samples, only the 100VEG sample differed from the others (*p* ≤ 0.05). Oleic acid (C18:1n9c) was found in higher concentration in the hybrid formulations and 100ANI (*p* ≥ 0.05) and in lower concentration in 100VEG (*p* ≤ 0.05) (Table 5). It belongs to the omega‐9 family and is found in vegetable oils (olive, avocado, peanut, sesame, and sunflower) and in animal sources (fats, egg yolks, and fish). It contributes to the maintenance of cardiovascular health, protects brain and nerve function, and reduces inflammatory processes (Wang et al. 2025).

In polyunsaturated foods, the highest concentrations were recorded in the 75VEG25ANI formulation, followed by 100VEG (*p* ≥ 0.05), with lower values for the others (*p* ≤ 0.05) (Table 5). Linoleic acid (C18:2n6c) was the most abundant omega‐6 fatty acid, a major component of sesame oil, and is also found in other oilseeds, nuts, legumes, eggs, meats, and dairy products. It plays important roles in lipid metabolism and cellular signaling, contributing to normal physiological functions and overall health. (Lima et al. 2024; Feng et al. 2025).

Omega‐3 fatty acids were detected at low levels, as expected, as their main sources are fish, flaxseed, soybean, and canola oils, walnuts, and chia seeds (Patted et al. 2024). Alam et al. (2025) found very low levels of *ω* − 3 fatty acids in beef, hybrid, and plant‐based burgers.

A *trans*‐fatty acid, elaidic acid (C18:1n9t), was identified at low concentrations in beef and hybrid burgers—a *trans*‐isomer of oleic acid—which can raise LDL cholesterol levels. Its presence, at 2%–5% in beef, justifies its detection in formulations containing meat and its absence in the plant sample (Pećina and Ivanković 2021). Flores et al. (2025) also detected this compound in pork burgers.

The use of sesame oil increased PUFA levels, which may explain the greater susceptibility to lipid oxidation (Islam et al. 2023). Although saturated fatty acids were reduced, this represents a technological limitation in terms of oxidative stability, highlighting a trade‐off between lipid composition and product stability. The effects of substitutions on the lipid fraction have been presented; the following section addresses their impact on the burgers’ metabolic profile.

### 3.5 Metabolic Profile

**FIGURE 1:**
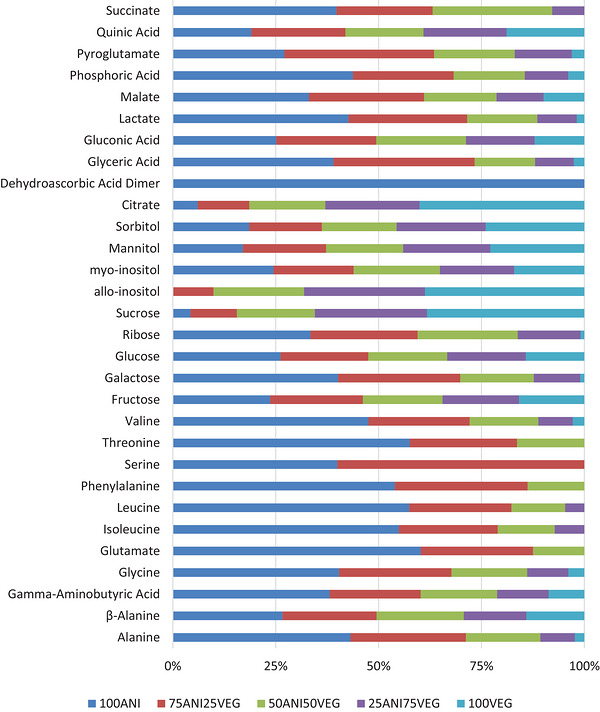
Relative metabolic content of burger formulations with partial or total replacement of beef by vegetable ingredients. 100ANI: 100% animal burger; 75ANI25VEG: 75% animal/25% vegetable burger; 50ANI50VEG: 50% animal/50% vegetable burger; 25ANI75VEG: 25% animal/75% vegetable burger; 100VEG: 100% vegetable burger.

Thirty metabolites belonging to the classes of amino acids, organic acids, sugars, and polyalcohols were identified, highlighting important metabolic differences among the formulations according to the degree of meat replacement (Figure 1). In general, formulations with higher meat proportions showed greater concentrations of essential amino acids, reducing sugars, and organic acids associated with energy metabolism. In contrast, formulations with higher levels of plant‐based ingredients presented increased levels of sucrose and specific polyols. These findings demonstrate that partial or complete meat replacement significantly alters not only the chemical composition but also the nutritional and technological profiles of hybrid and plant‐based burgers.

The higher concentrations of essential amino acids, such as leucine, isoleucine, valine, phenylalanine, and threonine observed in formulations with higher meat content (Figure 1), are particularly relevant from a nutritional perspective, as these compounds are associated with muscle protein synthesis, metabolic regulation, antioxidant activity, and neurocognitive functions (Ribes et al. 2024). Similarly, greater levels of glycine, glutamate, β‐alanine, and gamma‐aminobutyric acid (GABA) were detected in meat‐rich formulations, suggesting a higher contribution of metabolites associated with antioxidant, anti‐inflammatory, and neurotransmission‐related mechanisms (Dandare et al. 2020; Sarlo and Holton 2021; Imenshahidi and Hossenzadeh 2022; Ostfeld et al. 2024). These findings are consistent with the centesimal composition results (Table 3), which showed progressively lower protein contents as meat replacement increased.

Although plant‐based proteins may contribute substantially to protein intake, they generally have lower concentrations of certain essential amino acids than animal proteins, potentially affecting biological value and protein digestibility depending on the formulation composition (Lv et al. 2022; Surya Ulhas et al. 2023). In this context, the progressive reduction of amino acids observed in formulations with greater meat replacement reinforces the importance of complementary protein strategies in hybrid and plant‐based products to improve nutritional quality and amino acid balance (Dolganyuk et al. 2023). Similar behavior was reported by Cutroneo et al. (2023), who observed higher amino acid concentrations and greater protein digestibility in beef burgers than plant‐based analogues.

Beyond nutritional implications, the metabolic modifications may also influence technological and sensory properties. Formulations with higher meat content showed greater concentrations of reducing sugars such as glucose and fructose (Figure 1), compounds directly involved in Maillard reactions during cooking. These reactions contribute to the formation of melanoidins and volatile compounds responsible for characteristic meat aroma, flavor, and browning (Santos et al. 2025). Therefore, the simultaneous reduction in amino acids and reducing sugars in formulations with greater plant substitution may partially explain the differences observed in instrumental color parameters (Table 6) and texture profile (Table 8).

**TABLE 6:** Color parameters of burger formulations with partial or total replacement of beef by vegetable ingredients (mean ± SD).

Raw formulations	Parameters				
	L*	a*	b*	C*	*h*°
**100ANI**	27.35 ± 1.80^d^	9.88 ± 1.15^a^	23.64 ± 2.76^a^	24.01 ± 2.50^a^	66.41 ± 2.03^b^
**75ANI25VEG**	26.95 ± 1.27^d^	7.16 ± 0.53^bc^	17.18 ± 1.70^b^	18.60 ± 1.55^b^	66.35 ± 1.39^b^
**50ANI50VEG**	31.80 ± 0.90^c^	7.43 ± 0.49^b^	16.81 ± 1.05^b^	18.38 ± 1.07^b^	66.14 ± 1.34^b^
**25ANI75VEG**	36.69 ± 1.33^b^	6.16 ± 0.25^c^	16.58 ± 0.55^b^	17.69 ± 0.54^b^	69.61 ± 0.86^a^
**100VEG**	44.03 ± 2.10^a^	6.22 ± 0.68^bc^	17.07 ± 0.90^b^	18.17 ± 1.06^b^	70.03 ± 1.17^a^
**Fried formulations**	**Parameters**				
**100ANI**	29.98 ± 1.79^a^	8.57 ± 2.26^ab^	21.20 ± 2.50^a^	23.59 ± 2.68^a^	72.30 ± 2.07^a^
**75ANI25VEG**	23.64 ± 4.31^b^	11.40 ± 4.02^a^	20.99 ± 6.29^a^	19.35 ± 2.60^ab^	69.24 ± 2.57^a^
**50ANI50VEG**	17.05 ± 1.32^c^	10.03 ± 4.18^ab^	19.92 ± 2.54^a^	20.88 ± 4.50^ab^	70.26 ± 2.96^a^
**25ANI75VEG**	20.05 ± 4.10^bc^	5.31 ± 0.56^b^	15.56 ± 1.98^a^	16.44 ± 2.04^b^	71.10 ± 0.92^a^
**100VEG**	17.33 ± 2.37^c^	6.23 ± 1.61^b^	19.33 ± 3.35^a^	19.04 ± 3.39^ab^	70.45 ± 2.27^a^

*Note*: 100ANI: 100% animal burger; 75ANI25VEG: 75% animal/25% vegetable burger; 50ANI50VEG: 50% animal/50% vegetable burger; 25ANI75VEG: 25% animal/75% vegetable burger; 100VEG: 100% vegetable burger. *n* = 3 repetitions. Means followed by the same letters in the same column do not differ from each other by Tukey's test (*p* ≥ 0.05). L*: lightness; a*: tonality of red/green; b*: tonality of yellow/blue; C*: saturation or intensity of color; *h*°: hue or tone angle.

The higher concentrations of sucrose, mannitol, sorbitol, and allo‐inositol in formulations with greater plant‐based substitution (Figure 1) are likely associated with the vegetable ingredients incorporated into the formulations. In addition to their nutritional relevance, these compounds may contribute to modifications in water retention, structural organization, and texture stability. This behavior may help explain the lower cooking loss and shrinkage, as well as the higher yields observed in formulations with higher levels of meat replacement (Table 7). Furthermore, polyols such as mannitol and sorbitol present low glycemic indices, which may represent a nutritional advantage for consumers seeking products with lower glycemic impact (Trivedi et al. 2023).

**TABLE 7:** Cooking loss, shrinkage, and yield of burger formulations with partial or total replacement of beef by vegetable ingredients (mean ± SD).

Formulations	Parameters		
	Cooking Loss (%)	Shrinkage (%)	Yield (%)
**100ANI**	38.64 ± 1.51^a^	22.63 ± 1.33^a^	61.36 ± 1.51^d^
**75ANI25VEG**	10.15 ± 1.06^b^	15.04 ± 1.42^b^	89.85 ± 1.06^c^
**50ANI50VEG**	9.89 ± 1.30^b^	5.91 ± 1.07^c^	90.11 ± 1.30^c^
**25ANI75VEG**	4.01 ± 0.99^d^	2.99 ± 1.40^d^	95.99 ± 0.99^a^
**100VEG**	7.60 ± 1.27^c^	1.89 ± 0.23^d^	92.40 ± 1.27^b^

*Note*: 100ANI: 100% animal burger; 75ANI25VEG: 75% animal/25% vegetable burger; 50ANI50VEG: 50% animal/50% vegetable burger; 25ANI75VEG: 25% animal/75% vegetable burger; 100VEG: 100% vegetable burger. *n* = 3 repetitions. Means followed by the same letters in the same column do not differ from each other by Tukey's test (*p* ≥ 0.05).

Among the identified organic acids, phosphoric, glyceric, gluconic acids, lactate, and malate were more abundant in formulations with higher meat proportions, whereas citrate showed higher concentrations in 100VEG (Figure 1). These compounds are associated with flavor modulation, antioxidant mechanisms, and metabolic pathways related to amino acid and carbohydrate metabolism (Zhang et al. 2021; Li et al. 2022; Shi et al. 2022; Wang, Feng, et al. 2024; Wang, Marks, et al. 2024). The reduction of these metabolites in formulations with greater plant substitution further reinforces the metabolic differences between animal‐ and plant‐based matrices.

The metabolic alterations observed in the present study highlight important nutritional and technological trade‐offs associated with meat replacement. Although incorporating plant ingredients and sesame oil improved the lipid profile by reducing saturated fatty acids and increasing PUFAs (Table 5), progressive meat replacement simultaneously reduced the concentration of metabolites associated with muscle metabolism, biological protein quality, and Maillard reaction potential. Therefore, hybrid formulations, particularly 50ANI50VEG, may represent a more balanced alternative, combining improved technological properties, such as reduced cooking loss and acceptable texture characteristics (Tables 7 and 8), with partial preservation of the nutritional and metabolic attributes associated with meat‐based products.

**TABLE 8:** Texture profile of burger formulations with partial or total replacement of beef by vegetable ingredients (mean ± SD).

Formulations	Parameters				
	Hardness (N)	Elasticity (mm)	Cohesiveness	Gumminess (N)	Chewiness (N mm)
**100ANI**	30.32 ± 12.93^b^	0.74 ± 0.03^a^	0.48 ± 0.06^a^	14.96 ± 7.37^b^	11.13 ± 5.46^b^
**75ANI25VEG**	53.71 ± 10.41^a^	0.74 ± 0.03^a^	0.47 ± 0.06^a^	25.78 ± 7.34^a^	19.15 ± 6.00^a^
**50ANI50VEG**	35.30 ± 5.61^b^	0.63 ± 0.04^b^	0.41 ± 0.03^b^	14.36 ± 2.61^b^	9.06 ± 1.80^b^
**25ANI75VEG**	20.68 ± 4.39^c^	0.44 ± 0.05^c^	0.29 ± 0.03^c^	6.15 ± 1.74^c^	2.78 ± 0.97^c^
**100VEG**	8.52 ± 2.32^d^	0.19 ± 0.03^d^	0.16 ± 0.03^d^	1.40 ± 0.57^d^	0.28 ± 0.17^c^

*Note*: 100ANI: 100% animal burger; 75ANI25VEG: 75% animal/25% vegetable burger; 50ANI50VEG: 50% animal/50% vegetable burger; 25ANI75VEG: 25% animal/75% vegetable burger; 100VEG: 100% vegetable burger. *n* = 3 repetitions. Means followed by the same letters in the same column do not differ from each other by Tukey's test (*p* ≥ 0.05).

Abbreviations: mm, millimeter; N, Newton.

Overall, the metabolic profile results demonstrate that meat replacement affects multiple dimensions of product quality simultaneously, including nutritional value, technological performance, sensory‐related properties, and metabolic functionality. These findings reinforce the importance of integrated formulation strategies that balance sustainability objectives with nutritional adequacy, oxidative stability, technological quality, and consumer acceptance in hybrid and plant‐based meat products (Xie et al. 2024).

### 3.6 Appearance and Instrumental Color Parameters

**FIGURE 2:**
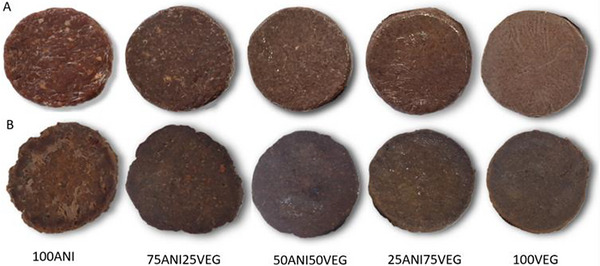
Formulations of raw (A) and fried burgers (B) with partial or total replacement of beef by vegetable ingredients. 100ANI: 100% animal burger; 75ANI25VEG: 75% animal/25% vegetable burger; 50ANI50VEG: 50% animal/50% vegetable burger; 25ANI75VEG: 25% animal/75% vegetable burger; 100VEG: 100% vegetable burger.

The addition of freeze‐dried açaí pulp produced similar color shades and significantly affected both burger appearance (Figure 2) and instrumental color parameters (Table 6).

The value of L* indicates lightness, ranging from 0 (black) to 100 (white). In raw samples, differences were observed (*p* ≤ 0.05), except between 100ANI and 75ANI25VEG (*p* ≥ 0.05). After frying, L* increased in 100ANI (*p* ≤ 0.05), and 75ANI25VEG differed from the others except 25ANI75VEG (*p* ≥ 0.05) (Table 6). The higher L* in hybrid and vegetable raw samples may be related to the use of sesame oil as a fat substitute. Bakhsh et al. (2022) observed the same condition in lactoferrin‐analog burgers and red yeast rice.

For the a* coordinate, which indicates hue along the red‐green axis, the 100ANI formulation raw differed from the others (*p* ≤ 0.05). It showed a higher value, attributed to the myoglobin present in the meat. Da Silva et al. (2025) added jabuticaba and strawberry extracts to pork burgers and observed the same behavior.

After frying, the 75ANI25VEG sample differed from 25ANI75VEG and 100VEG (*p* ≤ 0.05) (Table 6). The reduction in the a* value in the 100ANI formulation can be explained by myoglobin degradation, as observed by Vu et al. (2022).

Regarding the b* coordinate, which indicates hue along the yellow–blue axis, the raw burgers were similar to each other (*p* ≥ 0.05), differing only from 100ANI (*p* ≤ 0.05). After frying, there was no difference between the formulations (*p* ≥ 0.05) (Table 6), similar to the research by Janardhanan et al. (2023). The authors obtained 20.79 in the animal sample, 21.12 in the hybrid, and 22.33 in the vegetable sample, values that are close to those reported in the present study.

The value of C* indicates the intensity of the color. In raw samples, the control differed from the others (*p* ≤ 0.05). After frying, 100ANI and 25ANI75VEG differed (*p* ≤ 0.05). For hue angle (*h*°) in raw samples, 100ANI, 75ANI25VEG, and 50ANI50VEG were similar, as were 25ANI75VEG and 100VEG (*p* ≥ 0.05). Frying increased *h*°, with no differences among burgers (*p* ≥ 0.05) (Table 6).

Values between 0° and 90° indicate shades of red to yellow, expected for meat products, as well as positive values for a* and b* (King et al. 2023). In the present study, this coloration can be attributed to the presence of myoglobin (meat), isoflavones (soy protein), and anthocyanins (açaí) (Han et al. 2024; Yang et al. 2024). Heat treatment degrades anthocyanins, resulting in orange tones that increase b* (yellowing) and reduce a* (redness) (Zhao et al. 2024).

Bellucci et al. (2022) obtained approximate values of C* and *h*° in raw pork burgers with erythorbate (18.38; 61.26) and açaí extract (17.43; 56.62). Given these results and the growing demand for natural food dyes, açaí anthocyanins stand out as a promising alternative, offering attractive color and nutritional benefits and thereby enhancing product acceptance.

The cooking temperature (150°C), lower than typical conditions (200–220°C), may have limited Maillard reactions and the development of aroma, color, and flavor, affecting comparability with commercial products. On the other hand, these reactions can also generate compounds with potentially harmful health effects (Silva Barbosa Correia et al. 2024). Despite this, the recommended internal temperature (74–78°C) was reached, with an observed value of 75°C, allowing the comparison of the formulations’ technological properties.

### 3.7 Cooking Loss, Shrinkage, and Yield

In the cooking loss, a significant difference was observed between the formulations (*p* ≤ 0.05), except for 75ANI25VEG and 50ANI50VEG (*p* ≥ 0.05). The rate decreased as meat was replaced with vegetable ingredients, except for 25ANI75VEG, which had a lower percentage (Table 7). Janardhanan et al. (2023) reported the greatest loss in animal‐origin samples (25.06%), followed by hybrid (4.93%) and vegetable (0.99%).

Zhang et al. (2024) observed that cooking loss increased with increasing temperature, being higher in beef burgers (32.60% to 40.0%) than in vegetable analogues (17.80% to 27.00%). These results indicate that soy protein reduces losses due to its greater water‐retention capacity. Similarly, Lin and Barbut (2025) found that increasing soy protein levels in chicken meat progressively reduced cooking loss.

The shrinkage rate differed among formulations (*p* ≤ 0.05), except between 25ANI75VEG and 100VEG (*p* ≥ 0.05), and decreased as meat substitution increased (Table 7). Chin et al. (2024) reported that increasing the replacement of meat with plant‐based ingredients (0%, 25%, 50%, 75%, and 100%) reduced shrinkage and cooking loss, findings similar to those in this study.

After frying, the 100ANI and 75ANI25VEG samples showed greater deformations, whereas the others maintained their cylindrical shape, as shown in Figure 2. This effect is associated with the greater release of liquids from the meat, resulting from the shrinkage of the protein network due to the denaturation of collagen, myosin, and actin. In contrast, soy proteins were already denatured before cooking, conferring greater structural stability. Rice and cassava starch also contribute to water retention in hybrid products and vegetables (Vu et al. 2022).

Regarding yield, there was a significant difference between the formulations (*p* ≤ 0.05), except 75ANI25VEG and 50ANI50VEG (*p* ≥ 0.05) (Table 7). Yields increased as meat was replaced by plant products, except for 25ANI75VEG, which showed higher yields, a behavior similar to that observed in cooking weight loss. Thus, it can be inferred that partial replacement of meat with vegetable ingredients, particularly up to 75%, improved yield and reduced cooking loss under the evaluated conditions.

Cho et al. (2023) found that vegetable burgers made with high‐moisture analogues had the highest yield (95.29%), followed by the control (89.07%) and low‐moisture (77.49%), indicating that moisture content influences mass retention after preparation.

Yield is an important indicator of meat product behavior during cooking, as it reflects the protein matrix's ability to retain water and fat; thus, lower yields are associated with greater exudation losses during heating. Plant‐based products tend to exhibit greater fluid retention and, consequently, higher yields. These results are desirable because they reduce losses, maintain the final weight, and preserve the product's shape, contributing to a better perception of quality and freshness by consumers (Tabarestani et al. 2024).

Reduced cooking loss and shrinkage, along with higher yield, are associated with compositional changes, particularly the incorporation of plant ingredients with greater water retention capacity. These effects reflect modifications in the protein matrix, influencing water and fat retention during cooking and highlighting a balance between composition, structure, and technological performance (Hollweg et al. 2024), as further evidenced by the microstructure analysis presented in the following section.

### 3.8 Microstructure

**FIGURE 3:**
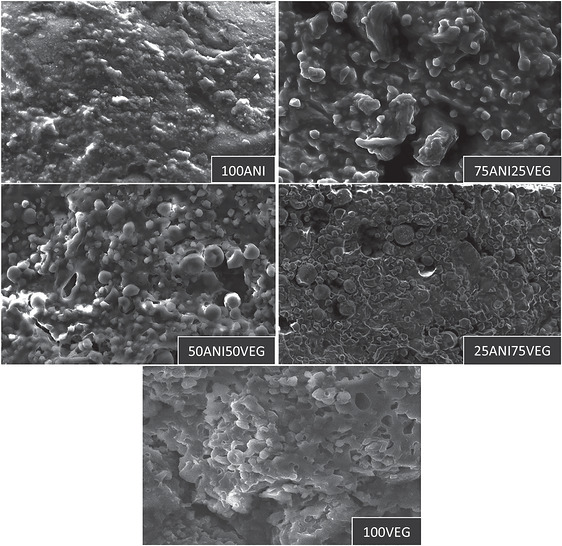
Scanning electron microscopy images of burger formulations with partial or total replacement of beef by vegetable ingredients. 100ANI: 100% animal burger; 75ANI25VEG: 75% animal/25% vegetable burger; 50ANI50VEG: 50% animal/50% vegetable burger; 25ANI75VEG: 25% animal/75% vegetable burger; 100VEG: 100% vegetable burger. Magnification: 2000×.

The animal‐based burger (100ANI) has a homogeneous structure and a smooth surface (Figure 3). A dense, cohesive protein matrix is evident, with fat globules distributed throughout the structure, a common characteristic of restructured products, such as burgers. Similar results were obtained by Nascimento et al. (2024) in beef burgers.

The burger's morphology was affected by replacing beef with a plant‐based alternative. The burger with 25% meat replacement (75ANI25VEG) showed larger gaps, greater porosity, and greater surface irregularity than the others (Figure 3). The addition of powdered ingredients as meat substitutes promoted interactions between animal and plant proteins, resulting in a heterogeneous distribution of components in the product structure due to the expansion of components such as water, fat, or air (Li et al. 2025).

As the percentage of plant‐based meat substitution increased, the network structure became denser. In the 50ANI50VEG formulation, spheres with regular shapes and sizes and fewer voids are observed compared to 75ANI25VEG. In 25ANI75VEG, there is a reduction in the coarse, heterogeneous structure compared to 75ANI25VEG (Figure 3).

According to Bee Chi et al. (2024), the higher the percentage of plant ingredients, the fewer cavities will be observed. For the authors, the ideal beef replacement rate with a vegetable mixture is 50%.

The plant‐based burger (100VEG) has an irregular surface layer (Figure 3). Plant ingredients lack the water‐holding capacity and fibrous texture present in animal muscle, which makes it difficult to obtain analogues with structural characteristics similar to those of meat products. In addition, processing conditions and the properties of the food matrix directly influence the product's structural, rheological, functional, textural, and sensorial properties (Ahmad et al. 2024).

Overall, microstructural changes explain the differences in technological and textural properties among formulations. The denser protein matrix in the animal‐based burger is associated with higher protein content and greater cohesion. At the same time, increased porosity and structural reorganization in plant‐based formulations reflect interactions between ingredients with distinct functional properties (Wei et al. 2023), as further evidenced by the texture parameters discussed in the following section.

### 3.9 Texture Profile

Regarding hardness, the maximum force required to compress the food between the molar teeth, there was a significant difference between the formulations (*p* ≤ 0.05), except 100ANI and 50ANI50VEG, which were statistically similar (*p* ≥ 0.05). The hardness was significantly higher in the burger with 25% meat replacement by vegetables (75ANI25VEG) and lower in the 100% vegetable formulation (100VEG) (Table 8).

The increase in hardness is generally associated with the strengthening of cross‐links in the protein matrix, which possibly occurred in the 75ANI25VEG formulation. Non‐flesh proteins can act as water and fat binders, promoting a less cohesive structure, as seen in the 25ANI75VEG and 100VEG formulations, which have higher percentages of vegetable ingredients (Zhou et al. 2022). Flores et al. (2025) observed that hybrid burgers were softer than pork burgers. Similarly, Souppez et al. (2025) found that plant‐based burgers are less tough than beef burgers.

Elasticity and cohesiveness differed among formulations (*p* ≤ 0.05), except between 100ANI and 75ANI25VEG (*p* ≥ 0.05), and decreased with meat replacement (Table 8). Elasticity measures the sample's ability to recover after deformation, whereas cohesiveness evaluates the resistance of internal bonds to rupture (Guerrero et al. 2025). Penalver et al. (2025) observed that plant‐based burgers have less elasticity and cohesiveness than hybrids and meat, attributed to the absence of connective tissue. Higher values of these parameters indicate lower deformation and greater structural consistency (Mirzan et al. 2025).

Gumminess was highest in 75ANI25VEG and lowest in 100VEG, with differences among samples (*p* ≤ 0.05) except between 100ANI and 50ANI50VEG (*p* ≥ 0.05) (Table 8). This variable refers to the energy required to grind the food until it is fully prepared for swallowing (Laili and Sofyan 2024). Mabrouki et al. (2024) observed that the analogous burgers exhibited lower gumminess than the traditional sample.

Chewiness differed among hybrid formulations (*p* ≤ 0.05); however, 25ANI75VEG and 100VEG were similar (*p* ≥ 0.05), and 100ANI and 50ANI50VEG were also similar (*p* ≥ 0.05) (Table 8). This parameter reflects the combination of gumminess and elasticity and defines the amount of energy required to chew the burgers (Zhao et al. 2024).

The highest chewiness was observed in 75ANI25VEG, and the lowest in 100VEG (Table 8). Janardhanan et al. (2023) reported similar relationships among hardness, gumminess, and chewiness, with the hybrid sample showing the highest values, followed by the animal and vegetable samples.

Replacing 50% of beef was effective, as 50ANI50VEG did not differ from 100ANI in hardness, gumminess, or chewiness (Table 8), a result supported by microstructure analysis (Figure 3). In contrast, 75ANI25VEG showed higher values, associated with greater porosity, wider gaps, and a more irregular surface.

Texture differences are associated with compositional and structural changes due to meat replacement. Reduced protein content and altered amino acid composition likely weakened the protein network, whereas plant ingredients influenced water retention and matrix organization, affecting texture parameters (Hollweg et al. 2024).

Given that burgers are susceptible to lipid oxidation, leading to undesirable changes in color, aroma, flavor, and texture, these structural differences reinforce the importance of evaluating stability parameters, such as pH, lipid oxidation, cooking loss, shrinkage, and yield, during 120 days of frozen storage to ensure product quality and stability.

### 3.10 Physicochemical and Physical Stability

#### 3.10.1 PH and Lipid Oxidation

The pH analysis results indicated that all samples are classified as low‐acidity (pH > 4.50). There was a statistical difference (*p* ≤ 0.05) between the formulations over the evaluated period, except on the last day of storage, when the 25ANI75VEG and 100VEG hamburgers did not differ (*p* ≥ 0.05). There was a statistical difference (*p* ≤ 0.05) as a function of storage period, formulation, and their interaction. The greatest reduction in values was observed on the last day of storage (Table 9).

**TABLE 9:** Potential of hydrogen (pH) and thiobarbituric acid reactive substances (TBARS) of burger formulations with partial or total replacement of beef by vegetable ingredients during frozen storage (−18°C ± 1°C) for 120 days (mean ± standard deviation).

Storage days	Formulations				
	100ANI	75ANI25VEG	50ANI50VEG	25ANI75VEG	100VEG
	**pH**				
**0**	5.65 ± 0.01^eA^	5.77 ± 0.01^dA^	5.85 ± 0.07^cA^	6.26 ± 0.02^bA^	6.44 ± 0.01^aA^
**30**	5.61 ± 0.04^eB^	5.75 ± 0.03^dAB^	5.85 ± 0.02^cA^	6.22 ± 0.01^bB^	6.36 ± 0.02^aB^
**60**	5.62 ± 0.03^eAB^	5.72 ± 0.03^dB^	5.83 ± 0.02^cAB^	6.24 ± 0.02^bAB^	6.37 ± 0.01^aB^
**90**	5.61 ± 0.01^eB^	5.73 ± 0.03^dAB^	5.83 ± 0.03^cAB^	6.22 ± 0.02^bB^	6.33 ± 0.04^aC^
**120**	5.50 ± 0.02^dC^	5.65 ± 0.06^cC^	5.78 ± 0.03^bB^	6.18 ± 0.01^aC^	6.19 ± 0.02^aD^
	**TBARS—mg MDA kg^−1^**				
**0**	0.85 ± 0.03^eE^	1.31 ± 0.05^dC^	1.39 ± 0.03^cC^	1.57 ± 0.03^bB^	2.45 ± 0.04^aB^
**30**	1.22 ± 0.08^eB^	1.61 ± 0.06^dB^	2.02 ± 0.05^cB^	2.25 ± 0.04^bA^	2.73 ± 0.08^aA^
**60**	1.01 ± 0.03^eD^	1.15 ± 0.05^dD^	1.39 ± 0.07^cC^	1.58 ± 0.03^bB^	1.96 ± 0.03^aC^
**90**	1.30 ± 0.04^eA^	1.61 ± 0.03^dB^	2.11 ± 0.05^cA^	2.26 ± 0.02^bA^	2.44 ± 0.04^aB^
**120**	1.11 ± 0.03^dC^	1.71 ± 0.04^bA^	2.15 ± 0.03^aA^	1.61 ± 0.02^cB^	1.79 ± 0.12^bD^

*Note*: 100ANI: 100% animal burger; 75ANI25VEG: 75% animal/25% vegetable burger; 50ANI50VEG: 50% animal/50% vegetable burger; 25ANI75VEG: 25% animal/75% vegetable burger; 100VEG: 100% vegetable burger. *n* = 3 repetitions. Means followed by the same lowercase letters in the same row and means followed by the same uppercase letters in the same column do not differ from each other by Tukey's test (*p* ≥ 0.05).

Abbreviation: MDA, malondialdehyde.

The results of lipid oxidation determination indicated a statistical difference (*p* ≤ 0.05) between the formulations during storage, except on the last day, when the 75ANI25VEG and 100VEG burgers did not differ (*p* ≥ 0.05). There was a statistical difference (*p* ≤ 0.05) as a function of storage period, formulation, and their interaction. The values fluctuated during storage (Table 9).

Gotardo et al. (2025) evaluated red propolis extract (1800 and 3600 mg kg) as an antioxidant replacement for sodium erythorbate (500 mg kg) in lamb burgers frozen for 120 days. They observed pH between 5.59 and 5.80, close to the value obtained, and TBARS levels ranging from 0.6 to 6.0 mg MDA kg^−1^, higher than the maximum value reported in the present study.

During storage, TBARS values remained below the reported limit (2.50 mg MDA kg^−1^); however, results should be interpreted with caution due to possible interference from anthocyanins. Thus, oxidative stability and antioxidant effectiveness cannot be conclusively confirmed, although both additives were associated with pH stability.

#### 3.10.2 Cooking Loss, Shrinkage, and Yield

The 100ANI formulation showed the highest cooking loss, differing statistically from the others throughout the storage period (*p* ≤ 0.05); however, it was the only one to show a reduction at 120 days relative to the initial value. The 25ANI75VEG burger showed the lowest loss values during storage, except on the 90th day, when the 100VEG sample showed the lowest value. There was a statistical difference (*p* ≤ 0.05) as a function of storage period, formulation, and their interaction. The formulation 50ANI50VEG stands out positively, as it showed lower variation at 120 days relative to the initial value (Table 10).

**TABLE 10:** Cooking loss, shrinkage, and yield of burger formulations with partial or total replacement of beef by vegetable ingredients during frozen storage (−18°C ± 1°C) for 120 days (mean ± standard deviation).

Storage days	Formulations				
	100ANI	75ANI25VEG	50ANI50VEG	25ANI75VEG	100VEG
	**Cooking loss (%)**				
**0**	38.48 ± 1.55^aA^	9.95 ± 1.03^bB^	9.64 ± 1.22^bB^	3.90 ± 0.98^dC^	7.71 ± 1.62^cB^
**30**	38.00 ± 0.88^aA^	13.51 ± 2.12^bcA^	14.62 ± 0.96^bA^	9.16 ± 0.60^dAB^	12.89 ± 0.61^cA^
**60**	30.56 ± 1.72^aB^	12.05 ± 1.43^bA^	10.40 ± 1.07^bcB^	10.05 ± 0.70^cA^	11.75 ± 0.98^bA^
**90**	30.18 ± 1.93^aB^	11.70 ± 0.88^bAB^	9.36 ± 0.98^cB^	9.41 ± 0.81^cAB^	7.57 ± 0.98^dB^
**120**	31.49 ± 1.88^aB^	12.34 ± 1.05^bA^	10.00 ± 1.23^cdB^	8.59 ± 0.69^dB^	11.74 ± 1.33^bcA^
	**Shrinkage (%)**				
**0**	22.29 ± 1.69^aA^	14.48 ± 1.61^bA^	6.18 ± 1.23^cA^	2.65 ± 1.27^dBC^	1.83 ± 0.76^dB^
**30**	19.00 ± 2.21^aB^	7.93 ± 1.70^bB^	6.93 ± 1.23^bA^	2.21 ± 0.73^cC^	2.39 ± 0.59^cB^
**60**	19.05 ± 1.95^aB^	7.21 ± 1.70^bBC^	6.63 ± 1.26^bA^	3.87 ± 0.58^cAB^	2.23 ± 1.68^cB^
**90**	13.93 ± 0.77^aC^	5.52 ± 1.00^bC^	2.54 ± 1.28^cB^	2.38 ± 0.54^cC^	1.20 ± 0.51^dB^
**120**	14.82 ± 2.13^aC^	7.59 ± 2.13^bBC^	3.70 ± 1.57^cB^	3.98 ± 1.30^cA^	5.30 ± 1.55^bcA^
	**Yield (%)**				
**0**	61.52 ± 1.55^dB^	90.05 ± 1.03^cA^	90.36 ± 1.22^cA^	96.10 ± 0.98^aA^	92.29 ± 1.62^bA^
**30**	62.00 ± 0.88^dB^	86.49 ± 2.12^bcB^	85.38 ± 0.96^cB^	90.84 ± 0.60^aBC^	87.11 ± 0.61^bB^
**60**	69.44 ± 1.72^cA^	87.95 ± 1.43^bB^	89.60 ± 1.07^abA^	89.95 ± 0.70^aC^	88.25 ± 0.98^bB^
**90**	69.82 ± 1.93^dA^	88.30 ± 0.88^cAB^	90.64 ± 0.98^bA^	90.59 ± 0.81^bBC^	92.43 ± 0.98^aA^
**120**	68.51 ± 1.88^dA^	87.66 ± 1.05^cB^	90.00 ± 1.23^abA^	91.41 ± 0.69^aB^	88.26 ± 1.33^bcB^

*Note*: 100ANI: 100% animal burger; 75ANI25VEG: 75% animal/25% vegetable burger; 50ANI50VEG: 50% animal/50% vegetable burger; 25ANI75VEG: 25% animal/75% vegetable burger; 100VEG: 100% vegetable burger. *n* = 3 repetitions. Means followed by the same lowercase letters in the same row and means followed by the same uppercase letters in the same column do not differ from each other by Tukey's test (*p* ≥ 0.05).

The highest shrinkage rates were also observed in the 100ANI burger, which differed statistically from the others during storage (*p* ≤ 0.05). The 100VEG burger recorded the lowest values during storage, except on the 30th day, when the 25ANI75VEG sample showed the lowest rate, and on the last day, when the 50ANI50VEG product showed the lowest shrinkage. There was a statistical difference (*p* ≤ 0.05) as a function of storage period, formulation, and their interaction. At the end of storage, the shrinkage rate of 100ANI, 75ANI25VEG, and 50ANI50VEG burgers decreased compared to the first day of analysis (Table 10).

The lowest yield percentages were recorded in the 100ANI formulation, which differed from the others (*p* ≤ 0.05); however, it was the only one to show an increase at 120 days compared to the initial value. The 25ANI75VEG burger showed the highest yield, except on the 90th day, when the 100VEG burger had the highest average value. There was a statistical difference (*p* ≤ 0.05) as a function of storage period, formulation, and their interaction. At the end of storage, it was verified that the hybrid samples 50ANI50VEG and 25ANI75VEG showed the highest yield, followed by the vegetable product (Table 10).

Vu et al. (2022) also recorded greater cooking loss and shrinkage in traditional hamburgers. Ball et al. (2021) incorporated vegetable proteins (oats, peas, and rice) into beef burgers and reported lower yields in the control formulation compared with the hybrids.

Hybrid and plant‐based products exhibit greater fluid retention than meat, resulting in better yield, less shrinkage, and lower cooking losses (Hollweg et al. 2024). Therefore, it is found that one of the main advantages of partially replacing meat with vegetable ingredients is the improvement of technological properties.

Overall, the results indicate that hybrid formulations improve technological performance and maintain physicochemical stability during storage, although some limitations in oxidative assessment should be considered.

## 4 Limitations and Future Prospects

The determination of lipid oxidation by the TBARS method may have been affected by the anthocyanins in the açaí pulp, potentially interfering with the spectrophotometric readings and leading to an overestimation of the values; furthermore, the absence of complementary methods (peroxide index and hexanal) and corrections for the blank limits the robustness of this analysis. The absence of sensory evaluation, as required by the journal, due to the small number of evaluators (*n* = 9), limits inferences about acceptance attributes; additionally, the lower cooking temperature may have reduced Maillard reactions, affecting flavor and aroma development and hindering comparability with commercial products. Substituting meat reduces protein content and may limit the essential amino acid profile, potentially affecting the nutritional quality of the final product. The shelf life of the burgers was estimated at 120 days at freezing, as formulations with the highest plant proportion showed structural degradation after this period. Despite these limitations, the freeze‐dried acai was effective as a natural colorant, but additional application strategies can optimize phenolic compound retention and antioxidant activity; alternatively, it can be used only as a dye, keeping ascorbic acid as an antioxidant in all formulations. It is also suggested that protein supplementation be achieved through a combination of vegetable sources or fortification with limiting amino acids and that protein digestibility be evaluated. It is recommended to evaluate large‐scale production costs and commercial feasibility. Among the main limitations and barriers to the commercial introduction, the absence of specific regulation for plant products analogous to those of animal origin in Brazil stands out, which may generate legal uncertainty and hinder standardization and labeling.

## 5 Conclusion

Partial replacement of beef with plant‐based ingredients, combined with the use of sesame oil as a fat replacer and freeze‐dried açaí pulp as a natural antioxidant and colorant, resulted in burgers with modified technological and nutritional properties. Increasing levels of meat substitution led to reduced protein content and increased carbohydrate and energy values, while promoting an altered lipid profile with reduced saturated fatty acids, indicating the occurrence of nutritional trade‐offs. The 50% substitution level showed the most balanced performance, maintaining morphological and textural properties comparable to the control. However, limitations related to protein content, the potential quality of amino acids, and the increased susceptibility to lipid oxidation associated with sesame oil should be considered. Although the results indicate the technological viability of the formulations under the studied conditions, further studies involving sensory analysis, protein quality assessment, and complementary methods to evaluate lipid oxidation are needed to better understand the potential applications of these formulations. In this way, the study provides relevant information on the development of hybrid and vegetable burgers made with natural ingredients, while considering the limitations observed throughout the research. The oxidative stability results should be interpreted as preliminary due to methodological limitations associated with TBARS analysis in anthocyanin‐rich matrices.

## Author Contributions

**Sheyla Maria Barreto Amaral**: conceptualization, data curation, formal analysis, investigation, writing – original draft, methodology, validation. **Silmara de Fátima Silva Cavalcante**: investigation. **Janevane Silva de Castro**: investigation. **Luiz Alves Bitu**: investigation. **Felipe Sousa da Silva**: investigation. **Mairlane Silva de Alencar**: investigation. **Adriano Lincoln Albuquerque Mattos**: methodology, resources. **Ana Erbênia Pereira Mendes**: resources. **Paulo Henrique Machado de Sousa**: conceptualization, formal analysis, supervision, writing – review and editing, methodology. **Elisabeth Mary Cunha da Silva**: project administration, writing – review and editing, conceptualization, visualization, supervision.

## Conflicts of Interest

The authors declare no conflicts of interest.

## Data Availability

The data will be made available upon request.
